# Cytotoxic T Lymphocyte Antigen 4 Haploinsufficiency Presenting As Refractory Celiac-Like Disease: Case Report

**DOI:** 10.3389/fimmu.2022.894648

**Published:** 2022-07-22

**Authors:** Lauren V. Collen, Carlos Andres Salgado, Bin Bao, Erin Janssen, Dascha Weir, Jeffrey Goldsmith, Alan Leichtner, Nasim Sabery Khavari, Yael Gernez, Scott B. Snapper

**Affiliations:** ^1^ Division of Gastroenterology, Hepatology, and Nutrition, Department of Pediatrics, Boston Children’s Hospital and Harvard Medical School, Boston, MA, United States; ^2^ Division of Allergy, Immunology, and Rheumatology, Department of Pediatrics, Stanford University School of Medicine, Stanford, CA, United States; ^3^ Division of Immunology, Department of Pediatrics, Boston Children’s Hospital and Harvard Medical School, Boston, MA, United States; ^4^ Department of Pathology, Boston Children’s Hospital and Harvard Medical School, Boston, MA, United States; ^5^ Division of Pediatric Gastroenterology, Department of Pediatrics, Stanford University School of Medicine, Stanford, CA, United States

**Keywords:** CTLA4 haploinsufficiency, refractory celiac disease, celiac disease, primary immunodeficencies (PID), abatacept, CTLA4 deficiency, autoimmunity, case report

## Abstract

Primary immunodeficiency may present with treatment-refractory enteropathy. We present two patients with celiac/celiac-like disease diagnosed in early childhood and refractory to the gluten-free diet. One patient had features of multi-system autoimmunity, whereas the other had celiac-like disease as an isolated clinical finding. Both patients underwent genetic testing given disease refractoriness and were ultimately diagnosed with cytotoxic T lymphocyte antigen 4 (CTLA4) haploinsufficiency. They are both now in complete clinical and endoscopic remission on abatacept. CTLA4 haploinsufficiency has incomplete penetrance and significant phenotypic heterogeneity but should be considered in the differential diagnosis of refractory celiac/celiac-like disease, as treatment implications are significant.

## Introduction

Cytotoxic T lymphocyte antigen 4 (CTLA4) is a negative regulator of T cell–mediated immune responses. CTLA4 competes with CD28 to bind CD80 and CD86, two co-stimulatory molecules on the surface of antigen-presenting cells. CD28 is constitutively expressed on T cells and, upon binding to CD80 and CD86, delivers a positive signal, which results in T cell activation. CTLA4, in contrast, is primarily stored in intracellular vesicles and becomes upregulated in conventional T cells in the activated state. When CTLA4 is expressed on the T cell surface, it binds to CD80 and CD86 with higher affinity than CD28 and functions (1) to inhibit the positive signal from CD28 interaction with CD80/CD86 and (2) to deliver an independent negative signal, limiting the production of interleukin-2 (IL-2) and the proliferation and survival of T cells (1–5).

Heterozygous germline mutations in *CTLA4* in humans are characterized by a clinical phenotype with features of both autoimmunity and immunodeficiency, with increased risk for several malignancies (4, 6). This autosomal dominant syndrome has incomplete penetrance, with 67%–71% of mutation carriers having a clinical disease (4, 6). Clinical manifestations reported in a cohort of 133 CTLA4 insufficient subjects included hypogammaglobulinemia, lymphoproliferation, autoimmune cytopenias, respiratory, gastrointestinal, and neurologic features. Gastrointestinal features, when present, most commonly include diarrhea, autoimmune enteropathy, inflammatory bowel disease, and atrophic gastritis (6). Here, we present two novel cases of CTLA4 haploinsufficiency in which celiac/celiac-like disease refractory to the gluten-free diet (GFD) was the primary presenting feature prompting evaluation for immunodeficiency. While celiac disease has previously been reported in two patients with CTLA4 haploinsufficiency (6), this is the first detailed report highlighting both this uncommon clinical presentation and resolution of celiac-like enteropathy with abatacept. These cases highlight the value in considering genetic testing for primary immunodeficiencies in patients with refractory celiac disease, especially in this era of rapidly expanding knowledge of monogenic causes of disease and targeted therapeutic approaches.

## Case Report

### Patient 1

**Figure 1:**
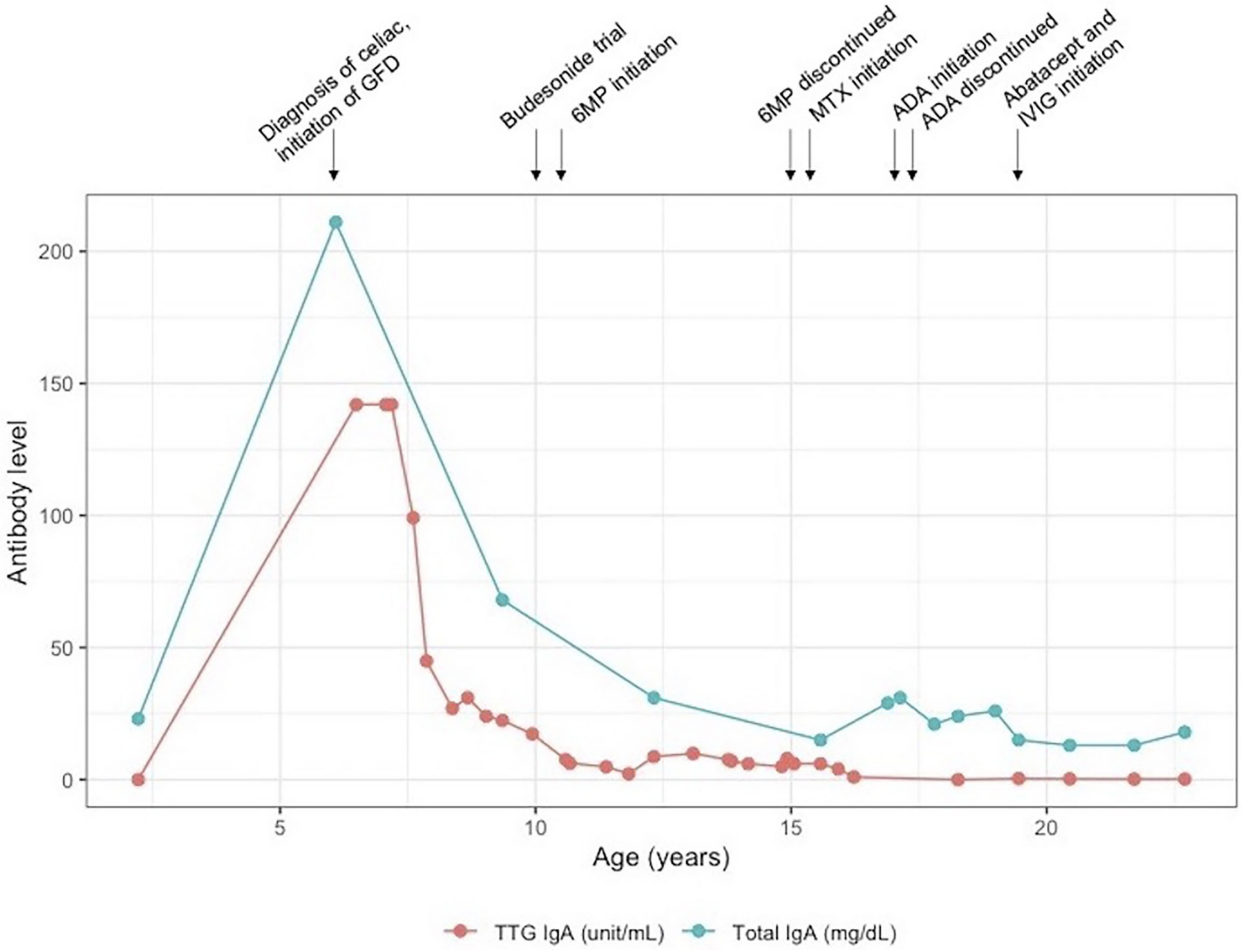
Laboratory trend in patient 1. 6MP, 6-mercaptopurine; ADA, adalimumab; GFD, gluten-free diet; IVIg, intravenous immunoglobulin; MTX, methotrexate.

**Figure 2:**
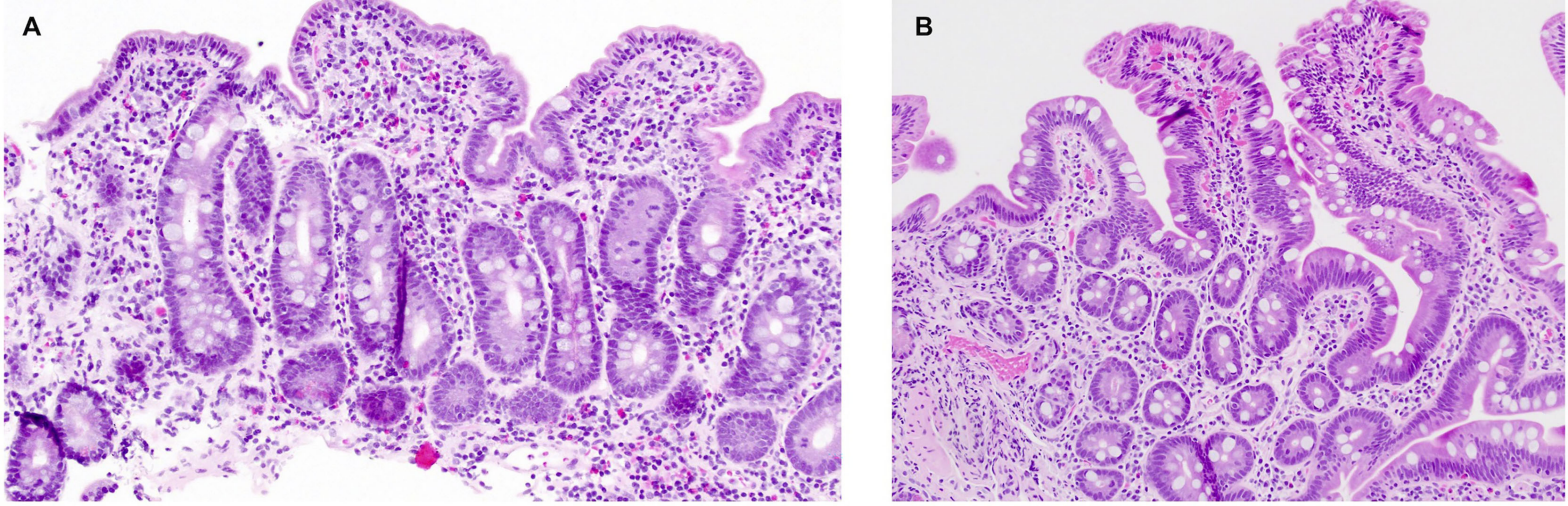
Histopathology findings in patient 1. **(A)** Duodenal mucosa with total villous atrophy, crypt hyperplasia, and patchy intraepithelial lymphocytosis, suggestive of celiac disease. **(B)** Normal duodenal mucosa, two years into treatment with abatacept.

Patient 1 is a 23-year-old female who presented in early infancy with diarrhea, not responsive to a trial of semi-elemental formula. She was referred to gastroenterology at age 6 for persistent non-bloody diarrhea with all growth parameters <5^th^ percentile for age and had laboratory and endoscopic evaluation consistent with celiac disease, with tissue transglutaminase (TTG) IgA > 142 EU/ml, endomysial antibody (EMA) 1:640 and esophagogastroduodenoscopy (EGD) with absent duodenal villi grossly and with increased intraepithelial lymphocytes on histopathology. She was initiated on a strict GFD. One year after her diagnosis, her TTG remained markedly elevated despite strict adherence to the GFD and careful efforts by her family and the medical team to identify possible sources of hidden gluten exposure. Over the subsequent decade, her TTG IgA slowly trended downward but remained positive **(** **Figure 1** **)**, and she continued to have diarrhea and secondary hypokalemia, iron deficiency anemia, and poor growth, intermittently requiring hospitalization for dehydration and electrolyte derangements in the setting of diarrheal output sometimes exceeding 6 L per day. She had serial EGDs and capsule endoscopies with atrophic mucosa, characterized by shortened or absent villi and scalloping of folds, at times affecting the mid and distal small bowel in addition to the duodenum. Histopathology continued to show changes consistent with celiac disease **(** **Figure 2A** **)**. The colon was grossly and histologically normal on multiple ileocolonoscopies. Stool cultures, including for *Salmonella* and *Campylobacter*; stool microscopy, including for *Cryptosporidium* and *Giardia*; and stool polymerase chain reaction (PCR) for norovirus 1 and norovirus 2 were negative on multiple occasions. She underwent various medication trials to complement treatment with GFD, including budesonide, 6-mercaptopurine (6MP), methotrexate, adalimumab, and prednisone. She had partial symptomatic and endoscopic improvement with 6MP, but use was limited by cytopenias. She had a significant symptomatic response with adalimumab, but this was subsequently discontinued after the development of drug-induced lupus following the fifth dose. She had an excellent but transient symptomatic response to prednisone.

**Figure 3:**
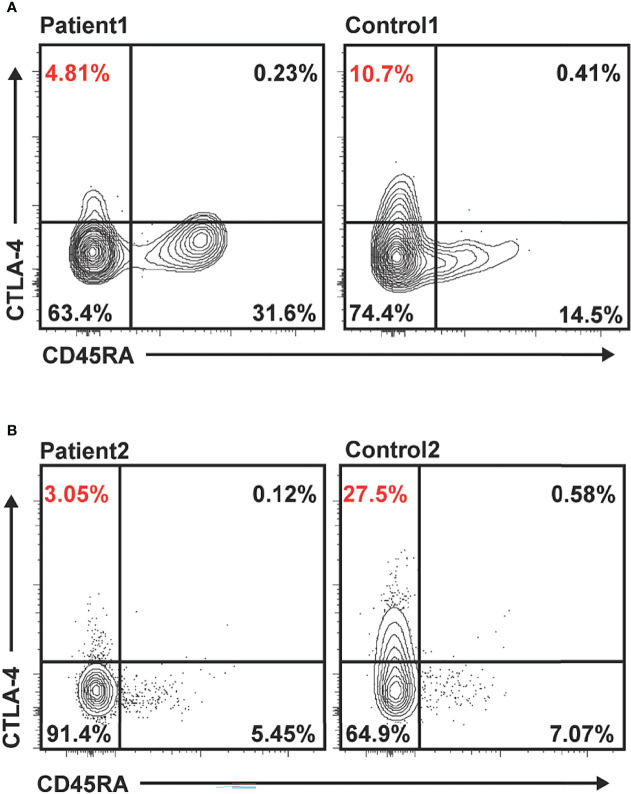
Reduced CTLA4 expression in unstimulated memory regulatory T cells (T_regs_) in patients 1 and 2 compared to healthy controls. Flow cytometry data gated on live/CD4^+^/Foxp3^+^. Representative expression of total CTLA4 in T_regs_ in patient 1 compared to healthy control **(A)** and patient 2 compared to healthy control **(B)**. Percentages shown in quadrants, with percentage CD45RA^−^ (memory) T_regs_ expressing CTLA4 highlighted in red.

While refractory celiac disease was patient 1’s primary complaint over her course, she was also afflicted with multiple other autoimmune processes, including Hashimoto’s thyroiditis (diagnosed age 6), Addison’s disease (diagnosed age 8), cytopenias (onset at age 8, prior to initiation of 6MP), and mild scalp psoriasis and possible lichen sclerosis (diagnosed age 16). She additionally had downtrending immunoglobulin levels over time and by age 19 fulfilled the criteria for common variable immunodeficiency (CVID) (7), based on low IgG (nadir 342 mg/dl, normal range 639–1,344 mg/dl), low IgA (**Figure 1**), and negative titers to varicella-zoster virus and mumps despite complete vaccination. Immunologic evaluation was also notable for low switched memory B cells (CD19^+^CD27^+^IgD^−^ 1.40%, normal range 8.3%–27.8%) and low unswitched memory B cells (CD19^+^CD27^+^IgD^+^ 4.90%, normal range 7.0%–23.8%). Summary of relevant immunologic evaluation can be found in **Table 1**. Research-based whole-exome sequencing was pursued at this time and confirmed previously unreported heterozygous *CTLA4* mutation (c. 457 + 2T > C), suggestive of CTLA4 haploinsufficiency. Immunophenotyping by flow cytometry confirmed diminished expression of CTLA4 on CD4^+^/Foxp3^+^/CD45RA^−^ memory regulatory T cells (T_regs_) (**Figure 3A** **;** **Supplementary Methods**). Assessment of CTLA4 expression was determined to be optimal in the CD4^+^/Foxp3^+^/CD45RA^−^ compartment in prior literature (8).

**Table 1:** Immunologic characteristics of patients at diagnosis of CTLA4 haploinsufficiency, prior to initiation of abatacept and IVIg^#^.

	Patient 1 value [reference range]	Patient 2 value [reference range]
Leukocytes (K cells/μL)	6.02 [5.52-9.29]	4.80 [4.00-11.00]
Neutrophils	3.65 [3.04-6.06]	3.9 [1.4-7.0]
Lymphocytes	1.18 [1.17-2.30]	***0.99*** [1.00-5.30]
**T cells**		
CD3+, absolute (cells/μL)	***933*** [1,000-2,600]	815 [800-3,500]
CD3+/CD4+, absolute (cells/μL)	675 [530-1,500]	457 [400-2,100]
CD3+/CD8+, absolute (cells/μL)	***204*** [330-1,100]	288 [200-1,200]
CD4+/CD45RA+, absolute, naïve T cells (cells/μL)	n/a	99 [no reference]
CD4+/CD45RO+, absolute, memory T cells (cells/μL)	n/a	348 [no reference]
CD4+CD25+CD127^low^ regulatory T cells (%)	***11.1*** [5.9-10.2]	n/a
CD4+Foxp3+ regulatory T cells (%)	n/a	10.48 [no reference]
**Natural killer cells**		
CD3-/CD56+ or CD16+, absolute (cells/μl)	111 [70-480]	n/a
CD3-/CD56+CD16+, absolute (cells/μl)	n/a	50 [no reference]
**B cells**		
CD19+, absolute (cells/μL)	130 [110-570]	***139*** [200-600]
CD19+CD27+IgD-, switched memory B cells (%)	***1.40*** [8.30-27.80]	2.3 [1.00-43.00]
CD19+CD27+IgD+, unswitched memory B cells	***4.90*** [7.00-23.80]	5.1 [2.00-28.00]
**Immunoglobulin antibodies (mg/dL)**		
IgG	***637*** [639-1,344]	***652*** [717-1,463]
IgA	***31*** [70-312]	114 [51-220]
IgM	69 [40-240]	***57*** [59-220]
IgG1	367 [240-1118]	n/a
IgG2	***64*** [124-549]	n/a
IgG3	74 [21-134]	n/a
IgG4	***<1*** [7-89]	n/a
**Soluble IL2 receptor**	***2977*** [45-1105 unit/mL]	***1496*** [175.30 - 858.20 pg/mL]
**Vaccination response**		
*Haemophilus influenza* type B (Polysaccharide Ribose Phosphate; ng/mL)	***<110*** [>1,000]	n/a
Diphtheria IgG (IU/mL)	n/a	0.26 [Positive]
Tetanus IgG (IU/mL)	1.21 [0.15-7.00]	0.53 [Positive]
Pneumococcal polysaccharide (mcg/mL)	Positive	***Negative***
Pneumococcus Type 1, IgG	***0.46*** [>1.3]	4.9 [≥2.3]
Pneumococcus Type 2, IgG	***0.94*** [>1.3]	***0.5*** [≥1.0]
Pneumococcus Type 3, IgG	***0.70*** [>1.3]	***1.4*** [≥1.8]
Pneumococcus Type 4, IgG	***1.26*** [>1.3]	1.1 [≥0.6]
Pneumococcus Type 5, IgG	6.57 [>1.3]	***1.7*** [≥10.7]
Pneumococcus Type 6B, IgG	***1.19*** [>1.3]	***4.6*** [≥4.7]
Pneumococcus Type 7F, IgG	***1.04*** [>1.3]	n/a
Pneumococcus Type 8, IgG	***1.19*** [>1.3]	***2.7*** [≥2.9]
Pneumococcus Type 9N, IgG	***0.86*** [>1.3]	n/a
Pneumococcus Type 9V, IgG	***0.18*** [>1.3]	3.4 [≥2.6]
Pneumococcus Type 10A, IgG	6.20 [>1.3]	***2.6*** [≥2.9]
Pneumococcus Type 11A, IgG	***0.87*** [>1.3]	***<0.4*** [≥2.4]
Pneumococcus Type 12F, IgG	2.16 [>1.3]	***<0.4*** [≥0.6]
Pneumococcus Type 14, IgG	6.76 [>1.3]	***0.5*** [≥7.0]
Pneumococcus Type 15B, IgG	2.54 [>1.3]	***0.8*** [≥3.3]
Pneumococcus Type 17, IgG	n/a	***4.2*** [≥17.8]
Pneumococcus Type 17F, IgG	5.64 [>1.3]	n/a
Pneumococcus Type 18C, IgG	***1.11*** [>1.3]	n/a
Pneumococcus Type 19A, IgG	15.29 [>1.3]	***1.6*** [≥17.1]
Pneumococcus Type 19F, IgG	6.36 [>1.3]	***2.6*** [≥15.0]
Pneumococcus Type 20, IgG	8.60 [>1.3]	n/a
Pneumococcus Type 20F, IgG	n/a	***0.7*** [≥1.3]
Pneumococcus Type 22F, IgG	2.28 [>1.3]	10.0 [≥7.2]
Pneumococcus Type 23F, IgG	***0.66*** [>1.3]	14.4 [≥8.0]
Pneumococcus Type 33F, IgG	2.06 [>1.3]	***<0.4*** [≥1.7]
**Celiac autoantibody titers**		
Tissue transglutaminase, IgA (U/mL)	0.4 [<6.9]	<1.0 [<8.0]
Deaminated gliadin peptide, IgG (EU/mL)	4 [<19]	<1.0 [<8.0]
Endomysial antibody, IgA (titer)	n/a	Negative

^#^ **Table 1** is limited to clinical laboratory evaluation.

n/a, not available.

Following confirmation of CTLA4 haploinsufficiency, the patient was initiated on intravenous abatacept (CTLA4-Ig). Her abatacept dose was uptitrated until she had complete resolution of diarrhea to a final dose of 800 mg every 4 weeks. She was simultaneously started on monthly intravenous immunoglobulin (IVIg) for CVID. She has been asymptomatic from a gastrointestinal standpoint with the resolution of cytopenias for over 2 years on a combination of abatacept and IVIg. Soluble IL-2 receptor (i.e., CD25) levels, which were characteristically high at the patient’s diagnosis of CTLA4 haploinsufficiency (9), were monitored on abatacept and trended downward to normal range. She has had one follow-up EGD and ileocolonoscopy, which were grossly and histologically normal (**Figure 2B**). She achieved normal adult height (36% ile) and a healthy body mass index (21.3 kg/m^2^). Her co-morbid autoimmune diseases are well controlled with levothyroxine, fludrocortisone, hydrocortisone, and topical dermatologic therapies. She has never had significant or life-threatening infections. A trial-off GFD was discussed with the patient but, ultimately, she opted to continue.

Following the patient’s diagnosis of CTLA4 haploinsufficiency, first-degree relatives were tested and it was confirmed that the disease-causing mutation was inherited from the patient’s father, whose history is remarkable for melanoma, but without a personal history of autoimmunity. Apart from this, the patient’s family history is notable for a healthy mother and brother and a paternal first cousin with type 1 diabetes mellitus.

Clinical characteristics of patient 1 are summarized in **Table 2**.

**Table 2:** Clinical characteristics of patients.

	Patient 1	Patient 2
Sex	Female	Female
Race/ethnicity	White	Hispanic
Age at initial presentation	<6 months	<12 months
Features at initial presentation	Chronic diarrhea, poor growth	Chronic diarrhea, vomiting, poor growth
Endoscopic features	Atrophic small bowel mucosa, with shortened or absent villi and scalloping of folds	Stomach with diffuse inflammation, characterized by friability, erythema, and mucus plaques. Duodenum with diffuse villous atrophy and scalloping.
Histologic features	Duodenal mucosa with total villous atrophy, crypt hyperplasia, and patchy intraepithelial lymphocytosis	Duodenal mucosa with villous blunting, increased intraepithelial lymphocytes and lamina propria expansion by a mixed inflammatory infiltrate. Stomach with moderate to severe chronic active gastritis.
Age at diagnosis of CTLA4 haploinsufficiency	18.8 years	14.0 years
CTLA4 mutation	c.457+2T>C	*De novo* heterozygous variant in CTLA4 in exon 1, c71_72del (p.Leu24Profs*35)
Medical history	- Celiac-like disease - Hashimoto’s thyroiditis - Addison’s disease - Cytopenias - Drug-induced lupus (adalimumab) - Mild scalp psoriasis - Lichen sclerosis	- Celiac-like disease - Pulmonary coccidiomycosis - Erythema nodosum - Avoidant restrictive food intake disorder - Depression
Family history	- Father with CTLA4 haploinsufficiency and melanoma. - Paternal first cousin with type 1 diabetes mellitus.	- Maternal grandmother with type 1 diabetes mellitus
Consanguinity	None	None
Treatments	- GFD (NR) - Budesonide (NR) - 6-mercaptopurine (cytopenias) - Methotrexate (NR) - Adalimumab (drug-induced lupus) - Prednisone (LOR) - Abatacept (remission) - IVIg	- GFD (NR) - Budesonide (NR) - Abatacept (remission) - IVIg
Outcome	Clinical and endoscopic remission on abatacept	Clinical and endoscopic remission on abatacept
Follow-up period, since diagnosis of CTLA4 haploinsufficiency	5.0 years	2.0 years

NR, non-response; LOR, loss of response; n/a, not available.

### Patient 2

Patient 2 is a 14-year-old female who initially presented with diarrhea, vomiting, and failure to thrive before 1 year of age. She was referred for gastroenterology consultation, and celiac serologies and endoscopy were performed and reported to be abnormal (results not available). She was diagnosed with celiac disease and started on a GFD. She was asymptomatic on GFD through age 12, although a retrospective review of her growth revealed a decrease in height-for-age from 50^th^ percentile to <3^rd^ percentile and a decrease in weight-for-age from 25^th^ percentile to <3^rd^ percentile over that time. Weight loss acutely worsened at age 12, when she developed chronic nausea, vomiting, and abdominal pain in the setting of treatment for lung coccidiomycosis associated with erythema nodosum. This patient is from Central Valley, California, an endemic coccidiomycosis region. Endoscopy and colonoscopy were repeated at this time and demonstrated persistent villous atrophy and increased intra-epithelial lymphocytes, suggestive of celiac disease but with negative celiac serologies. She required multiple admissions over the next 4 years for these symptoms and ultimately had such profound weight loss that a nasogastric tube was placed for initiation of formula feeding. Magnetic resonance (MR)-enterography showed no bowel wall thickening or features suggestive of inflammatory bowel disease. Chronic infection with enteropathogens including norovirus was excluded. During a disease flare, an EGD was notable for stomach with diffuse inflammation, characterized by friability, erythema, mucus plaques, and duodenum with diffuse villous atrophy and scalloping. Histopathology showed moderate to severe chronic active gastritis in the stomach and villous blunting, increased intraepithelial lymphocytes and lamina propria expansion by a mixed inflammatory infiltrate in the duodenum. Ileocolonoscopy was grossly normal, with histology notable for increased intraepithelial lymphocytes in the ileum. TTG IgA and EMA were negative. Human leukocyte antigen (HLA) DQ2 and DQ8 were also negative.

Primary immunodeficiency gene panel testing Invitae, San Francisco, USA was done to evaluate for monogenic causes of autoimmune enteritis. A previously unreported *de novo* heterozygous variant in *CTLA4* in exon 1, c71_72del (p.Leu24Profs*35) was identified, suggesting a diagnosis of autosomal dominant CTLA4 haploinsufficiency. This sequence change creates a premature translational stop signal in the *CTLA4* gene. This is expected to result in an absent or disrupted protein product and is predicted to be pathogenic. Immunophenotyping by flow cytometry confirmed absent expression of CTLA4 on memory T_regs_ (**Figure 3B**). Immunological laboratories demonstrated B cell lymphopenia (absolute CD19^+^ of 139/µl, normal 200–600), decreased memory B cells (absolute CD19^+^CD27^+^ of 9.6/µl, normal 50–200), mild hypogammaglobulinemia (IgG 580 mg/dl), and poor response to polysaccharide pneumococcal vaccine; see **Table 1**. The patient had no evidence of other autoimmune conditions, although atrophic gastritis has been considered, given histologic findings on EGD.

The patient was started on IVIg. Further immunosuppressive treatment decisions for this patient were complicated given her history of lung coccidiomycosis and continued residence in a Coccidioides-endemic region. At the time of her referral, she was no longer receiving antifungal therapy and did not have clinical or radiographic evidence of infection. The presence of a common heterozygous variant in *PLCG2* (p.Arg268Trp), not routinely reported in the diagnostic gene panel, was ruled out, as this specific variant was found in five of 66 patients with disseminated coccidiomycosis in one study (10). Ultimately, treatment with weekly subcutaneous abatacept was initiated for the treatment of celiac-like disease. Subcutaneous delivery was initially chosen specifically for its hypothetic decreased risk for infection compared with intravenous (unpublished). Soluble IL2R levels rapidly decreased, but the patient continued to have nausea, vomiting, and poor oral intake, prompting transition to intravenous abatacept and addition of oral budesonide. Patient 2 is now 6 months into abatacept treatment at a dose of 10 mg/kg every 2 weeks. She has tapered off budesonide. She is asymptomatic and her most recent EGD was grossly and histologically normal for the first time. During the treatment course, patient 2 has had an intermittent recurrence of her erythema nodosum, confirmed by skin biopsy. This prompted infectious evaluation, which included negative peripheral blood dimorphic fungal PCR, fungal cultures from skin and stomach biopsies negative for coccidiomycosis, and negative chest X-ray. Notably, erythema nodosum has been reported in one other patient with CTLA4 haploinsufficiency during treatment with sirolimus (6).

## Discussion

To our knowledge, this is the first detailed report of CTLA4 haploinsufficiency presenting with refractory celiac-like disease as the primary manifestation. These cases highlight the value of keeping a high index of suspicion for CTLA4 haploinsufficiency in patients with refractory celiac disease, especially when multiple autoimmune diseases or a suggestive family history are present. Importantly, case 2 highlights that CTLA4 haploinsufficiency can mimic refractory celiac disease, even in the absence of other features suggestive of immunodeficiency or autoimmunity.

Identification of CTLA4 haploinsufficiency has important implications for therapeutics and cancer risk. From a therapeutic standpoint, both cases are examples of life-changing responses to abatacept, a fusion protein that binds to CD80 and CD86 receptors on antigen-presenting cells (APCs), blocking interactions with CD28 and thereby inhibiting T cell activation. This precision medicine approach directly addresses the mechanism by which CTLA4 haploinsufficiency results in immune dysregulation. Abatacept is currently approved only for use in rheumatoid arthritis (RA) and, notably, both patients 1 and 2 required higher dosing than is typically used in RA to achieve a complete response. The largest report on therapeutic approaches to CTLA4 haploinsufficiency, which includes 123 patients, comments on responses to systemic and topical corticosteroids, abatacept, azathioprine, sirolimus, and tumor necrosis factor-α inhibitors in the subset of 74 patients (60%) with gastrointestinal involvement. Of these patients, nine of 74 used abatacept, and all nine had an initial clinical response, although two ultimately discontinued abatacept after diarrhea recurred. These nine patients notably used different dosing than reported in our patients (subcutaneous weekly dosing versus higher, less frequent intravenous dosing), and none of the nine patients who used abatacept were reported to have presented with celiac-like disease (11).

Hematopoietic stem cell transplant (HSCT) can be curative in patients with CTLA4 haploinsufficiency but carries its own risks and has not yet been indicated in the cases we present (4, 6, 11). In the report on treatment outcomes in CTLA4 haploinsufficiency by Egg et al, 18 patients underwent HSCT, of whom 13 were alive and cured and five had died of complications of transplant or primary disease at last follow-up (11). To our knowledge, there are no reports to inform the continuation of GFD in patients with celiac-like disease secondary to CTLA4 haploinsufficiency. Both patients continue on GFD at this time. Patient 1 was offered the option of discontinuing the GFD with monitoring for recurrence of symptoms, based on the hypothesis that abatacept might mitigate the immune dysregulation that propagates celiac enteropathy; however, her preference was to continue the GFD.

The diagnosis of CTLA4 haploinsufficiency also carries important implications for cancer risk, with increased risks for several cancers, in particular Epstein Barr Virus (EBV)-associated lymphoma and gastric adenocarcinoma. In a cohort of 131 *CTLA4* mutation carriers with phenotypic features, 17 (12.9%) had malignancies, including five with gastric adenocarcinoma, with age at gastric adenocarcinoma diagnosis ranging from 17 to 42 years (4). Currently, there are no guidelines for surveillance EGDs in this population, so we have continued EGDs in patient 1 at every 2 year intervals.

Beyond CTLA4 haploinsufficiency, the differential diagnosis in patients presenting with refractory celiac/celiac-like disease can include lipopolysaccharide-responsive and beige-like anchor (LRBA) deficiency resulting from *LRBA* mutations and CVID (12, 13). LRBA controls the lysosomal turnover of CTLA4 in T cells, ultimately impacting surface expression of CTLA4. LRBA deficiency thus overlaps phenotypically with CTLA4 haploinsufficiency, with shared features including enteropathy, hypogammaglobulinemia, and autoimmune cytopenias. LRBA deficiency has been reported in association with one case of autoimmune gastritis responsive to abatacept (14) and in another case of refractory celiac-like disease cured by HSCT (13). Enteropathy is also a well-described feature of CVID, with chronic diarrhea being the most common gastrointestinal manifestation. Endoscopic and histologic findings in the small bowel can resemble celiac disease, with duodenal villous atrophy and increased intraepithelial lymphocytes reported in 51% and 76% respectively of a cohort of patients with CVID and gastrointestinal symptoms, nearly all of whom underwent upper endoscopy (12). Most often, these patients had negative celiac serologies, with only three of 50 patients from this report positive for TTG, EMA, gliadin, or reticulin autoantibodies. GFD was introduced in 12 of the CVID patients with villous atrophy, with only two showing clinical improvement, both of whom were interestingly negative for celiac autoantibodies (12). Importantly, chronic infection with enteropathogens, including norovirus, can trigger chronic enteropathy with associated intestinal villous atrophy and diarrhea in patients with CVID (15), so exclusion of infectious etiologies of chronic diarrhea is a critical component of evaluation.

Collectively, these cases highlight the value of considering an underlying immunodeficiency in patients with refractory celiac or celiac-like disease. Celiac disease cannot be distinguished from enteropathy secondary to CTLA4 deficiency solely by histopathology and, therefore, genetic testing is essential to identify this as an etiology of refractory celiac-like disease. Specific circumstances in which genetic testing should be considered include celiac disease refractory to the GFD, refractory celiac-like disease with negative serologies, celiac/celiac-like disease in the setting of multi-system autoimmunity, and celiac/celiac-like disease associated with severe, atypical, or recurrent infections. Rapidly increasing knowledge around monogenic etiologies of enteropathy and expanding precision medicine approaches, such as abatacept and HSCT, have tremendous potential to impact the quality of life and burden of disease in patients who have traditionally been treatment refractory.

## Data Availability Statement

The original contributions presented in the study are included in the article/**Supplementary Material**. Further inquiries can be directed to the corresponding author.

## Ethics Statement

Ethical review and approval was not required for the study on human participants in accordance with the local legislation and institutional requirements. Written informed consent to participate in this study was provided by the participants’ legal guardian/next of kin. Written informed consent was obtained from the individual(s), and minor(s)’ legal guardian/next of kin, for the publication of any potentially identifiable images or data included in this article.

## Author Contributions

LC cared for patient 1, wrote the manuscript, and prepared figures. CS cared for patient 2 and wrote part of the manuscript. BB performed immunophenotyping by flow cytometry. JG prepared figures and reviewed the manuscript. EJ, DW, AL, and NK contributed to patient identification and patient care and reviewed the manuscript. YG and SS cared for patients and reviewed and critically revised the work. All authors contributed to the article and approved the submitted version.

## Funding

LC is supported by the National Institute of Diabetes and Digestive Kidney Diseases of the National Institutes of Health under [award number T32 DK007477]. SS is supported by the National Institute of Diabetes and Digestive Kidney Diseases of the National Institutes of Health under [award number P30DK03485 and RC2DK122532], the Wolpow Family Chair in IBD Treatment and Research, the Translational Investigator Service at Boston Children’s Hospital, and the Children’s Rare Disease Cohort (CRDC) Study.

## Conflict of Interest

SS declares the following interests: scientific advisory board participation for Pfizer, BMS, Lilly, IFM Therapeutics, Merck, and Pandion Inc; grant support from Pfizer, Novartis, and Takeda; consulting for Hoffman La Roche, Takeda, and Amgen.

The remaining authors declare that the research was conducted in the absence of any commercial or financial relationships that could be construed as a potential conflict of interest.

## Publisher’s Note

All claims expressed in this article are solely those of the authors and do not necessarily represent those of their affiliated organizations, or those of the publisher, the editors and the reviewers. Any product that may be evaluated in this article, or claim that may be made by its manufacturer, is not guaranteed or endorsed by the publisher.
