# Supplementary material for: Cytotoxic T Lymphocyte Antigen 4 Haploinsufficiency Presenting As Refractory Celiac-Like Disease: Case Report
Source: Front Immunol. 2022 Jul 22;13:894648. doi: 10.3389/fimmu.2022.894648 (PMC9352891; doi:10.3389/fimmu.2022.894648)
Supplement: Supplementary file 1 [file DataSheet_1.docx]

**Supplementary Methods**

**Flow cytometry.** Cryopreserved peripheral blood mononuclear cells (PBMCs) were thawed and resuspended in phosphate-buffered saline (PBS). For surface staining, cells were incubated with CD4 APC-H7 (Invitrogen), CD127 BV650 (clone A019D5; Biolegend), CD25 PE-594 (clone M-A251; BD), CTLA4 PE-Cy7 (clone BNI3; Biolegend), CD45RA PerCP-Cy5.5 (clone HI100, Biolegend), live/dead BV510 (Invitrogen) at 4°C for 30 minutes. Cells were fixed in Cytofix at 4°C for 30 minutes then Foxp3 fixation buffer at 4°C for 30 minutes. For intracellular staining, cells were permeabilized by incubation in Foxp3 perm buffer at 4°C for 20 minutes, then incubated with Foxp3 APC (clone PCH101; Invitrogen) and CTLA4 PE-Cy7 (clone BNI3; Biolegend) at room temperature for 60 minutes. Cells were fixed on Cytofix at 4°C for 30 minutes then re-suspended in FACS buffer. Cells were acquired on a BD LSRFortessa and the data were analyzed using FlowJo software (TreeStar). Assessment of total CTLA4 expression was performed on CD45RA-Foxp3+ T cell fraction in accordance with prior literature.^8^
